# ZIF‐8‐Based Nanodelivery of Tectorigenin for Alleviating Skeletal Muscle Atrophy via Inhibition of Oxidative Stress and Inflammation

**DOI:** 10.1002/jcsm.70418

**Published:** 2026-11-10

**Authors:** Xia Li, Yutong Wei, Lei Qi, Xiu Dai, Zhanzhan Li, Chuli Zhu, Xucheng Zhu, Yuntian Shen, Bingqian Chen, Xiaosong Gu, Hualin Sun, Xinlei Yao

**Affiliations:** ^1^ Jiangsu Key Laboratory of Tissue Engineering and Neuroregeneration, Key Laboratory of Neuroregeneration of Ministry of Education, Co‐Innovation Center of Neuroregeneration, Medical School of Nantong University Nantong University Nantong Jiangsu People's Republic of China; ^2^ Department of Emergency Medicine Affiliated Hospital of Nantong University Nantong Jiangsu People's Republic of China; ^3^ Department of Orthopedics Changshu Hospital Affiliated to Soochow University, First People's Hospital of Changshu City Changshu Jiangsu People's Republic of China

**Keywords:** protein homeostasis, skeletal muscle atrophy, tectorigenin, ZIF‐8

## Abstract

**Background:**

Skeletal muscle atrophy is a pathological condition characterized by a progressive loss of muscle mass and function. Its core mechanisms involve an imbalance between protein synthesis and degradation, caused by oxidative stress and inflammation. Tectorigenin (TG) shows therapeutic potential but suffers from poor solubility and bioavailability. This study developed a zeolitic imidazolate framework‐8 (ZIF‐8) nanocarrier for TG (ZIF‐8@TG) to enhance delivery efficacy against neurogenic muscle atrophy.

**Methods:**

ZIF‐8@TG nanoparticles were synthesized and characterized for size, drug loading, release profile and cytotoxicity. Their therapeutic effects were investigated in vitro using a nutrient deprivation‐induced C2C12 myotube atrophy model and in vivo using a sciatic nerve crush injury–induced mouse model of muscle atrophy. Immunofluorescence staining and western blotting were performed to assess the effects of ZIF‐8@TG on atrophic myotubes. Behavioural tests, immunofluorescence staining and western blotting were used to evaluate its impact on muscle atrophy in vivo. The underlying mechanisms were further explored through western blotting, electron microscopy, immunofluorescence staining and H&E staining.

**Results:**

ZIF‐8@TG nanoparticles (approx. 50 nm) exhibited pH‐responsive release and efficient cellular uptake. In vitro, nutrient deprivation reduced myotube diameter by 53% (*p* < 0.01), whereas ZIF‐8@TG treatment restored it to 92% of control levels, showing significantly better efficacy than free TG. In vivo, sciatic nerve injury decreased tibialis anterior muscle mass by 35% (*p* < 0.001) and mean myofiber cross‐sectional area (CSA) by 41% (*p* < 0.001). ZIF‐8@TG treatment significantly increased muscle mass to 74% and CSA to 93% of control values, whereas free TG resulted in non‐significant improvements. Additionally, ZIF‐8@TG improved motor function in mice following sciatic nerve crush. Mechanistically, the alleviation of skeletal muscle atrophy by ZIF‐8@TG may be associated with regulation of excessive activation of the ubiquitin‐proteasome system and autophagy‐associated changes, along with enhanced protein synthesis–related Akt/mTOR signalling pathway during sciatic nerve crush–induced muscle atrophy, as well as an association with reduced oxidative stress and inflammation.

**Conclusions:**

ZIF‐8@TG effectively delivers TG, alleviating muscle atrophy in vitro and in vivo. ZIF‐8@TG treatment is associated with reduced oxidative stress and inflammation, preserved protein homeostasis and improved functional recovery, offering a promising nanotechnology‐based therapy strategy for neurogenic skeletal muscle atrophy.

## 1 Introduction

Skeletal muscle, the largest organ, governs movement, posture, metabolism and thermogenesis [1]. Its atrophy, driven by nerve injury, chronic disease, ageing, malnutrition or disuse [2], stems from disrupted proteostasis [3] involving ubiquitin‐proteasome system (UPS) and the autophagy‐lysosomal pathway (ALP) overactivation and AKT pathway suppression [4, 5]. Oxidative stress and inflammation amplify this imbalance, creating a vicious cycle [6]. Although exercise therapy is widely regarded as the most effective intervention, its applicability is limited in populations unable to engage in active physical activity, including individuals with severe neuromuscular impairments or those confined to bed rest [3]. Consequently, there is a pressing need to develop targeted pharmacological therapies to combat skeletal muscle atrophy.

In recent years, natural products have garnered significant attention in the development of therapeutics for skeletal muscle atrophy [7]. Tectorigenin (TG), an isoflavone compound isolated from traditional Chinese medicinal herbs such as Radix Puerariae, exhibits a broad spectrum of pharmacological activities, including antioxidant, anti‐inflammatory and antidiabetic effects [8, 9]. Studies have demonstrated that TG improves insulin resistance in skeletal muscle by upregulating glucose transporter 4 expression via activation of the AMPK/MEF2 signalling pathway [10]. More importantly, TG exerts potent antioxidant effects in a CCl4‐induced rat model of liver injury [11]. TG has also been shown to effectively suppress the recruitment and activation of hepatic macrophages, thereby attenuating inflammatory responses [12]. These findings collectively indicate that TG holds considerable potential for mitigating oxidative stress and inflammation associated with skeletal muscle atrophy. It is worth noting that its direct therapeutic application is limited by inherent pharmacokinetic challenges, including poor water solubility, low bioavailability and rapid metabolism in vivo [13]. Thus, the development of strategies for the effective and stable delivery of TG is necessitated.

To overcome the delivery challenges associated with natural products, nanotechnology provides innovative solutions for efficient drug delivery. The zeolitic imidazolate framework‐8 (ZIF‐8) is a pH‐responsive nanocarrier with high surface area, tunable pores, facile functionalization and good biocompatibility, which remains stable at neutral pH but degrades rapidly in acidic inflammatory environments, enabling targeted drug release [14]. This unique property renders ZIF‐8 an ideal carrier for targeted drug delivery. Research has demonstrated that ZIF‐8 can efficiently encapsulate proteins, nucleic acids and small‐molecule drugs while protecting them from premature degradation [15]. Consequently, the development of ZIF‐8‐based nanodelivery systems not only enhances the physicochemical stability of therapeutic agents but also may enhance therapeutic efficacy.

Based on the aforementioned background, this study aims to develop a novel nanodelivery system, ZIF‐8@TG, by encapsulating the natural bioactive molecule TG into the ZIF‐8 framework. First, the successful synthesis and characterization of ZIF‐8@TG nanoparticles were confirmed. Subsequently, the protective effects of ZIF‐8@TG against neurogenic muscle atrophy were systematically evaluated in vitro and in vivo. The underlying molecular mechanisms were investigated across multiple pathways, including oxidative stress, inflammatory response, protein hydrolysis, protein synthesis and neuromuscular junction (NMJ) repair. This study not only presents an efficient delivery strategy to facilitate the application of TG but also provides a novel conceptual framework and experimental foundation for the development of multifunctional nanomedicines in the treatment of neurogenic skeletal muscle atrophy.

## 2 Methods

### 2.1 Synthesis of ZIF‐8 and ZIF‐8@TG

ZIF‐8 was synthesized by mixing Zn (NO_3_)_2_·6H_2_O and 2‐methylimidazole in methanol at room temperature for 24 h. For ZIF‐8@TG, TG powder (10 mg/mL) was added after pre‐nucleation, and the reaction continued for 24 h. The product was collected by centrifugation, washed and freeze‐dried (for further details, see Supporting Information). Supporting Information contains the encapsulation efficiency (EE%) and drug loading capacity (LC%), stability assessment and detailed protocols for the synthesis and in vivo biodistribution of Cy5‐TG and Cy5‐ZIF‐8@TG. The biosafety assessment is also included in the Supporting Information.

### 2.2 Preparation and Analysis of Release Samples

In vitro release of TG from ZIF‐8@TG was evaluated in PBS at pH 6.0 and 7.4 at 37°C with continuous shaking. At predetermined time points, aliquots were centrifuged, and the supernatant was collected for absorbance measurement at 266 nm. The cumulative release was calculated based on a standard calibration curve (for further details, see Supporting Information).

### 2.3 Scanning Electron Microscopy (SEM)

The morphologies of the ZIF‐8 and ZIF‐8@TG nanoparticles were observed by SEM (ES‐2030, Hitachi, Japan).

### 2.4 Fourier Transform Infrared (FTIR)

FTIR spectroscopy was performed on the ZIF‐8 and ZIF‐8@TG nanoparticles at room temperature using a Thermo Fisher Scientific (USA) instrument, with spectral data collected over the wavenumber range of 400–4500 cm^−1^.

### 2.5 X‐Ray Diffraction (XRD)

The crystallographic phases of ZIF‐8 and ZIF‐8@TG were examined by XRD. A Bruker D8 ADVANCE diffractometer was employed for this analysis, utilizing Cu Kα radiation (λ = 0.15418 nm) and operating at 40 kV and 40 mA. The data were collected over a 2θ range of 5°–50° with a scan rate of 5° per minute.

### 2.6 Dynamic Light Scattering (DLS)

The hydrodynamic diameter, polydispersity index (PI), and zeta potential of ZIF‐8 and ZIF‐8@TG were measured by zeta potential and particle size analyser (DLS, 90Plus, Brookhaven).

### 2.7 Cell Culture

C2C12 myoblasts were cultured in DMEM with 10% FBS and differentiated into myotubes in DMEM with 2% horse serum for 6 days. Differentiated myotubes were treated with HBSS containing 20 μg/mL ZIF‐8@TG, ZIF‐8 or TG for 12 h to assess effects on nutrient deprivation‐induced atrophy (for further details, see Supporting Information).

### 2.8 Intracellular Uptake

C2C12 cells were incubated with 20 μg/mL ZIF‐8@TG for 6 h, washed, fixed with glutaraldehyde and processed for transmission electron microscopy (TEM) observation (for further details, see Supporting Information).

### 2.9 In Vitro Cytotoxicity Assay

C2C12 cells were seeded in a 96‐well plate at 4000 cells/well. After 12 h of culture, different concentrations (0, 10, 20, 40 and 60 μg/mL) of ZIF‐8@TG were added to the 96‐well plate and incubated for 24 h and washed three times with PBS, and then 10‐μL CCK‐8 was added to each well. The cells were incubated in an incubator at 37°C for 1 h. The absorbance of CCK‐8 at 450 nm was measured by a microplate reader.

### 2.10 Animals and Sciatic Nerve Crush Models

All animal procedures were approved by the Institutional Animal Care Committee (Approval No. S20240225‐006). Adult male ICR mice (8 weeks, 30–32 g) were randomly assigned to experimental groups. Sciatic nerve crush was performed under anaesthesia using haemostatic forceps for 15 s (repeated twice). Treatment groups received intraperitoneal injections every 3 days: 5 mg/kg TG, 40 mg/kg ZIF‐8 or ZIF‐8@TG at 5, 10, 20 or 40 mg/kg. Notably, the 40 mg/kg ZIF‐8@TG dose contains approximately the same amount of TG as the 5 mg/kg free TG dose. The vehicle control received 10% DMSO in saline. Behavioural tests and data analyses were performed blinded. Tissues were collected 18 days post‐surgery (for further details, see Supporting Information).

### 2.11 Western Blot

Muscle tissues were homogenized in lysis buffer containing protease and phosphatase inhibitors. After centrifugation, protein concentrations were determined by BCA assay. Equal amounts of protein were separated by SDS‐PAGE, transferred to PVDF membranes, blocked and incubated with primary antibodies overnight at 4°C, followed by HRP‐conjugated secondary antibodies. Protein bands were visualized using enhanced chemiluminescence and quantified with ImageJ software (for further details, see Supporting Information).

### 2.12 Immunofluorescence Staining

Cells and muscle sections were fixed, permeabilized (cells only), blocked and incubated with primary antibodies overnight at 4°C, followed by fluorochrome‐conjugated secondary antibodies. Nuclei were counterstained with DAPI. Muscle sections were obtained from the mid‐belly of the tibialis anterior (TA) at comparable levels; two random fields per section from three animals per group were analysed. Images were captured using a fluorescence microscope (for further details, see Supporting Information).

### 2.13 Detection of ROS Levels

Fresh tissue samples were embedded in OCT and sectioned into 6‐μm‐thick slices. The sections were washed three times with PBS (5 min per wash), followed by incubation with a DHE probe in the dark at room temperature for 30 min. Afterwards, the sections were washed three times with PBS (5 min per wash) and mounted with an anti‐fade mounting medium containing DAPI.

### 2.14 Succinate Dehydrogenase (SDH) Staining

Fresh tissue samples were embedded in OCT and sectioned into 6‐μm‐thick slices. The sections were rinsed with pure water for 10 min and then incubated in the dark at room temperature for 30 min using a commercial SDH staining kit. After incubation, the dye was washed off with pure water, and the sections were mounted with glycerol and analysed by microscope.

### 2.15 Gait Analysis

The gait of mice was assessed using the CatWalk XT system (Noldus Information Technology). Each mouse was placed on the glass walkway and allowed to traverse it voluntarily. As the mouse walked, a high‐speed colour camera positioned beneath the walkway captured its footprints. The resulting recordings were analysed using the integrated CatWalk XT software to obtain parameters of the gait pattern. Each mouse completed three consecutive runs.

### 2.16 Thermal Sensitivity Test

Mice were placed on a hot plate and allowed to acclimate for 30 min. A heat stimulus was applied to the plantar surface of the hind paw, and the withdrawal latency was recorded, with a cut‐off time of 30 s. Each paw was tested three times at 15‐min intervals, and the average latency was calculated (for further details, see Supporting Information).

### 2.17 Inverted Grid Test

Mice were placed on a metal wire grid (10 × 14 cm, 2‐mm wire diameter) positioned 40 cm above a padded surface. The grid was gently inverted, and the hanging time was recorded, with a maximum of 90 s. Each mouse underwent three trials at 10‐min intervals, and the best performance was used for analysis (for further details, see Supporting Information).

### 2.18 Statistical Analysis

All data were analysed using GraphPad Prism 9 software and are presented as the mean ± SD. The Shapiro–Wilk test was used to assess the normality of data distribution (*p* > 0.05 consistent with normal distribution). Brown–Forsythe test for homogeneity of variances. For multiple comparisons among groups, one‐way analysis of variance (ANOVA) followed by Tukey's post hoc test was applied. A *p* value < 0.05 was considered statistically significant.

## 3 Results

### 3.1 Successful Construction of ZIF‐8@TG Nanodelivery System and Uptake of ZIF‐8@TG in C2C12 Cells

ZIF‐8@TG nanoparticles were synthesized by co‐assembling TG with Zn^2+^ and 2‐methylimidazole (2‐Mel), whereby TG was incorporated into the ZIF‐8 framework (Figure 1A). As revealed by SEM, ZIF‐8@TG nanoparticles exhibited a rhombic dodecahedron morphology, consistent with the typical structure of ZIF‐8, and displayed a uniform particle size distribution of approximately 50–60 nm (Figure 1B). FTIR spectra indicated that all characteristic absorption peaks of ZIF‐8 were retained in ZIF‐8@TG. Additionally, several distinctive absorption peaks of TG were observed at 820, 838, 1229, 1267, 1550 and 1629 cm^−1^, providing preliminary evidence for the successful incorporation of TG into the ZIF‐8 matrix (Figure 1C). Moreover, the x‐ray diffraction (XRD) patterns of ZIF‐8 and ZIF‐8@TG nanoparticles were nearly identical, indicating that the incorporation of TG did not disrupt the ordered crystalline framework of ZIF‐8, which maintained a high degree of crystallinity (Figure S1A). The encapsulation efficiency (EE%) of TG in the ZIF‐8 nanoparticles was determined to be 3.2%. The loading capacity (LC%) of TG in the ZIF‐8 nanoparticles was 9.15%. After loading TG, the zeta potential value of ZIF‐8@TG decreased, possibly due to the presence of hydroxyl groups of TG (Figure S1B). ZIF‐8@TG exhibited uniformity under dynamic light scattering (DLS), with no aggregation observed and a polydispersity index of 0.144 (Figure S1C,D). These results collectively confirm the successful loading of TG into the ZIF‐8 nanoparticles. We further examined the stability of ZIF‐8@TG and found that particles were still evident in ZIF‐8@TG in saline after 24 h (Figure S1E).

**FIGURE 1:**
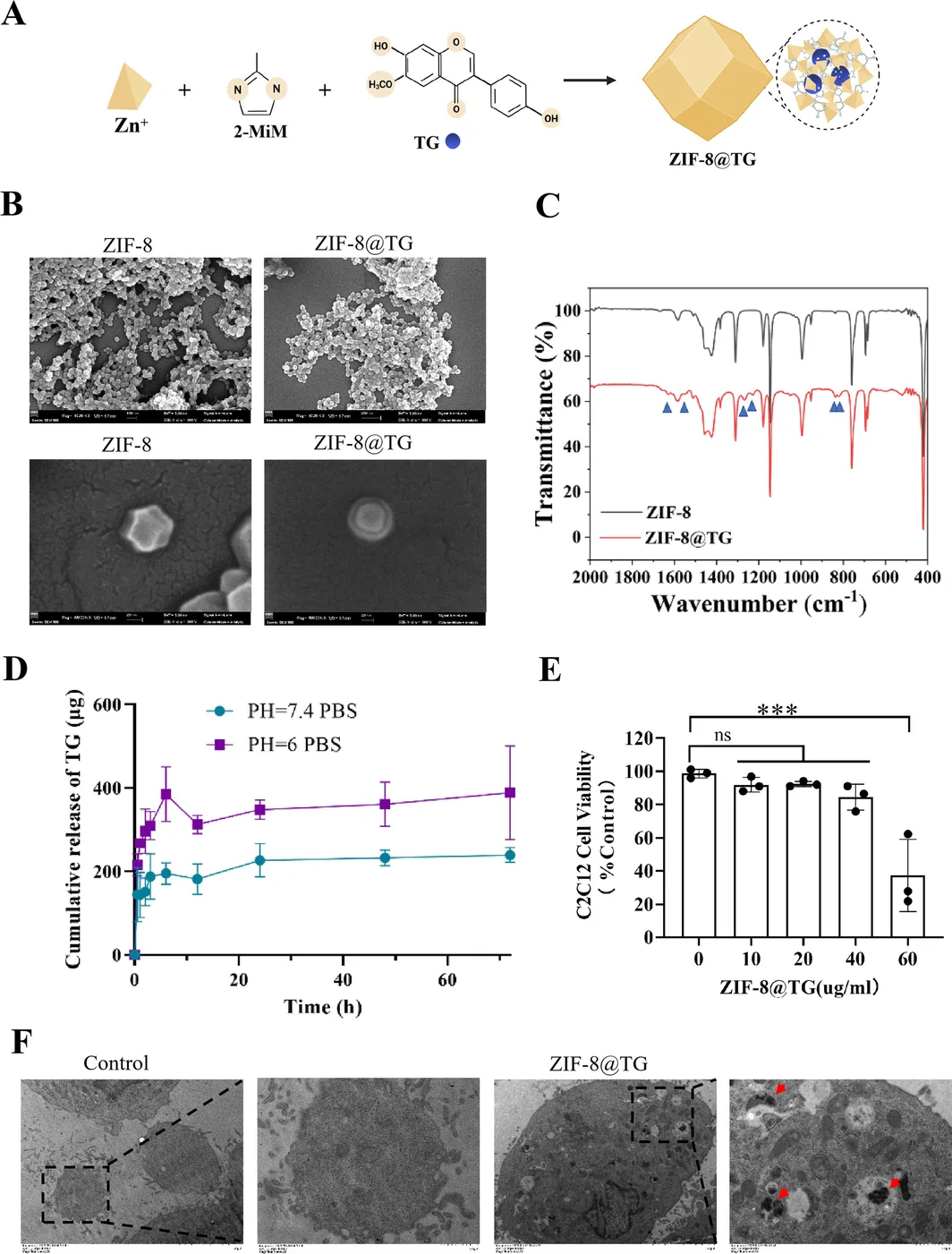
Characterization of ZIF‐8@TG and its cellular uptake. (A) Schematic illustration of the synthesis for ZIF‐8@TG nanoparticles. Created in BioRender (2026; https://BioRender.com/iwc7elm). (B) Representative scanning electron microscopy (SEM) images of ZIF‐8 and ZIF‐8@TG nanoparticles. Upper left: Multiple morphologies of ZIF‐8 nanoparticles, bars: 100 nm; lower left: Arrow indicates the morphology of a single ZIF‐8 nanoparticle, bars: 20 nm; upper right: Multiple morphologies of ZIF‐8@TG nanoparticles, bars: 200 nm; lower right: Arrow indicates the morphology of a single ZIF‐8@TG nanoparticle, bars: 20 nm. (C) Fourier transform infrared (FTIR) spectra of ZIF‐8 and ZIF‐8@TG nanoparticles. Blue arrows indicate characteristic absorption peaks of TG. (D) TG release from ZIF‐8@TG nanoparticles in phosphate buffer at pH 7.4 and pH 6.0. (E) Viability of C2C12 cells treated with various concentrations of ZIF‐8@TG for 24 h (*n* = 3). (F) TEM images showing the uptake of ZIF‐8@TG by C2C12 cells after 6 h of incubation with 20 μg·mL^−1^ ZIF‐8@TG. Scale bars: 5 and 1 μm. Data are presented as mean ± SD. ns = not significant, ***p < 0.001.

Drug release studies showed that TG was released more efficiently under acidic conditions than at physiological pH (Figure 1D). Subsequently, cytotoxicity assays confirmed that ZIF‐8@TG exhibited no significant cytotoxicity at concentrations below 40 μg·mL^−1^ in C2C12 cells (Figure 1E). Furthermore, TEM imaging revealed the presence of nanoparticles within the cytoplasm of C2C12 cells, confirming successful intracellular uptake of ZIF‐8@TG (Figure 1F). To visually observe its biodistribution in vivo, we used Cy5‐labelled ZIF‐8@TG nanoparticles. As shown in Figure S2A, at 24‐h post‐injection, Cy5‐labelled free TG exhibited only low accumulation in the injured (sciatic nerve crush side) muscle tissue; Cy5‐labelled ZIF‐8@TG nanoparticles showed significantly better accumulation in the injured muscle. Subsequently, ex vivo fluorescence imaging of the harvested ipsilateral muscles was performed. Compared with the Cy5‐labelled TG group, the Cy5‐labelled ZIF‐8@TG nanoparticles exhibited much stronger fluorescent signals (Figure S2B). These findings collectively demonstrate the successful fabrication of pH‐responsive ZIF‐8@TG nanoparticles capable of efficient cellular uptake and controlled TG release. Additionally, biosafety evaluation showed no significant pathological alterations in major organs (heart, liver, spleen, lung and kidney) following ZIF‐8@TG treatment (Figure S3). These results preliminarily indicate a favourable short‐term safety profile, although long‐term biosafety warrants further investigation.

### 3.2 ZIF‐8@TG Alleviates Nutrient Deprivation–Induced C2C12 Myotube Atrophy

We first explored the effects of ZIF‐8@TG nanoparticles on nutrient deprivation‐induced myotube atrophy. Myosin heavy chain (MHC) is a key structural component of myofilaments [16]. During muscle atrophy, activation of the STAT3/FoxO3 axis upregulates MAFbx, an E3 ubiquitin ligase, which promotes excessive ubiquitination and degradation of MHC [17]. MHC immunofluorescence staining showed that nutrient deprivation significantly reduced myotube diameter. Myotubes treated with ZIF‐8 exhibited an increase in diameter, but the difference was not statistically significant, and treatment with TG significantly increased myotube diameter, whereas ZIF‐8@TG treatment produced the most pronounced effect, restoring myotube diameter to a level nearly identical to that of the control group (Figure 2A,B). Western blot analysis further demonstrated that ZIF‐8@TG treatment significantly suppressed the reduction of MHC levels and attenuated the upregulation of both MAFbx and FoxO3a in C2C12 myotubes induced by nutrient deprivation (Figure 2C–F). These findings indicate that ZIF‐8@TG effectively alleviates nutrient deprivation–induced C2C12 myotube atrophy.

**FIGURE 2:**
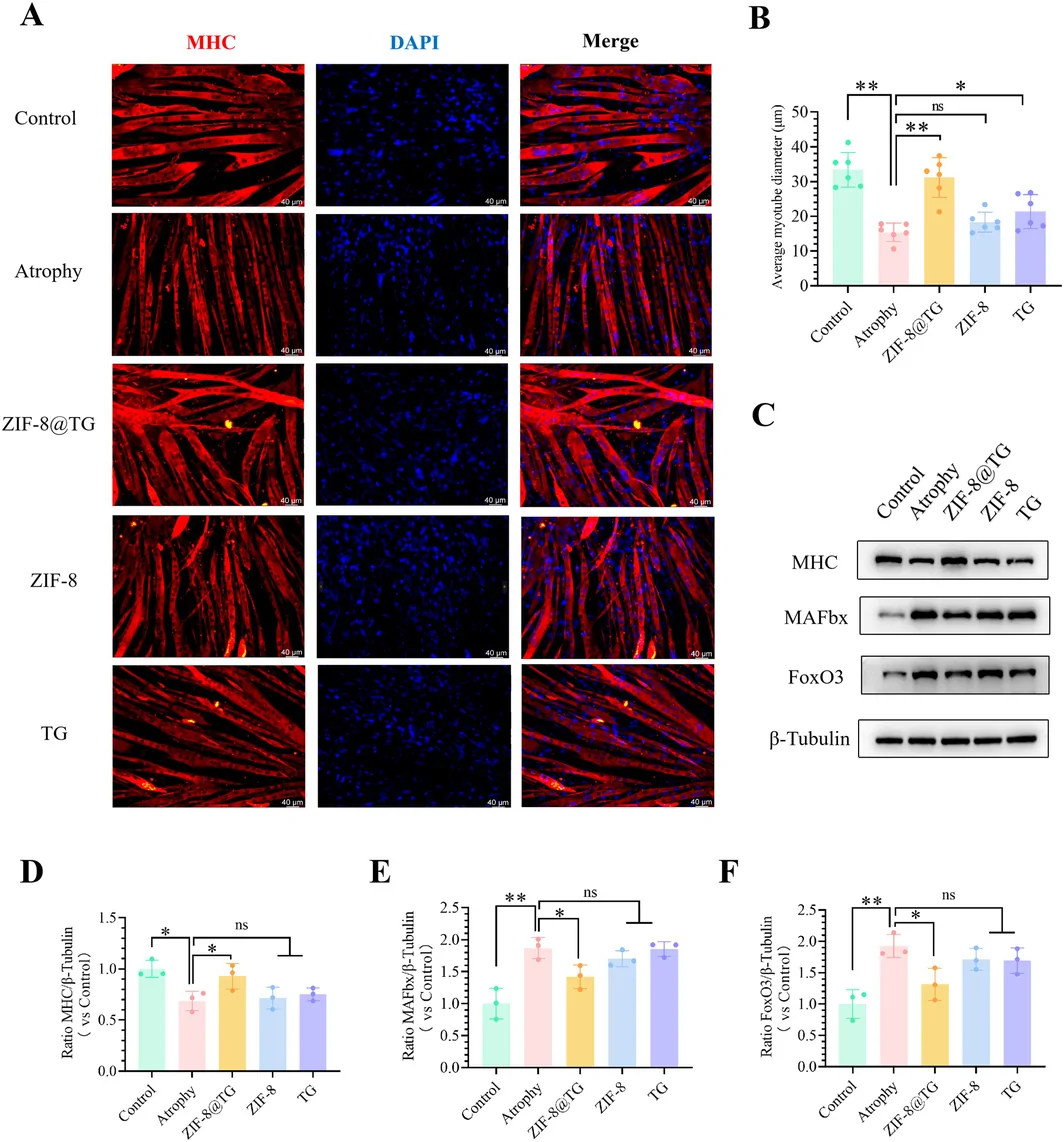
In vitro evaluation of ZIF‐8@TG for ameliorating myotube atrophy. (A) Representative MHC immunofluorescence images of C2C12 myotubes in different treatment groups. Scale bars: 40 μm. (B) Quantitative analysis of the average myotube diameter (*n* = 6). (C) Western blotting analysis of the levels of MHC, MAFbx and FoxO3a in differentiated C2C12 myotubes across treatment groups. (D–F) The quantitative analysis of MHC (*n* = 3), MAFbx (*n* = 3) and FoxO3a (*n* = 3) are presented as mean ± SD. ns = not significant, *p < 0.05, **p < 0.01, ***p < 0.001.

### 3.3 ZIF‐8@TG Improves Motor Function in Mice With Sciatic Nerve Crush

To evaluate the in vivo efficacy of ZIF‐8@TG, we established a mouse model of sciatic nerve crush–induced muscle atrophy. Gait analysis showed that the mice exhibited a reduced paw print area in crush group. Administration of ZIF‐8 or TG alone resulted in a slight improvement, but the differences have no significance (Figure 3A,B). Whereas treatment with ZIF‐8@TG led to a larger paw print area and a significant, dose‐dependent recovery of the sciatic functional index (SFI), with 40 mg·kg^−1^ being the optimal dose (Figure 3A,B). Consistently, hot plate and inverted grid tests revealed that mice in the crush group had a significantly increased paw withdrawal latency and a markedly decreased hanging time, indicating reduced pain sensitivity to thermal stimuli and impaired endurance/grip strength (Figure 3C,D). Whereas treatment with ZIF‐8 or TG alone shortened the paw withdrawal latency and increased the hanging time, the latter has no significance. Importantly, administration of ZIF‐8@TG significantly decreased the paw withdrawal latency and increased the hanging time in a dose‐dependent manner, with 40 mg/kg identified as the optimal dose (Figure 3C,D). These results demonstrate that ZIF‐8@TG treatment significantly improves motor function in mice, outperforming either ZIF‐8 or TG alone.

**FIGURE 3:**
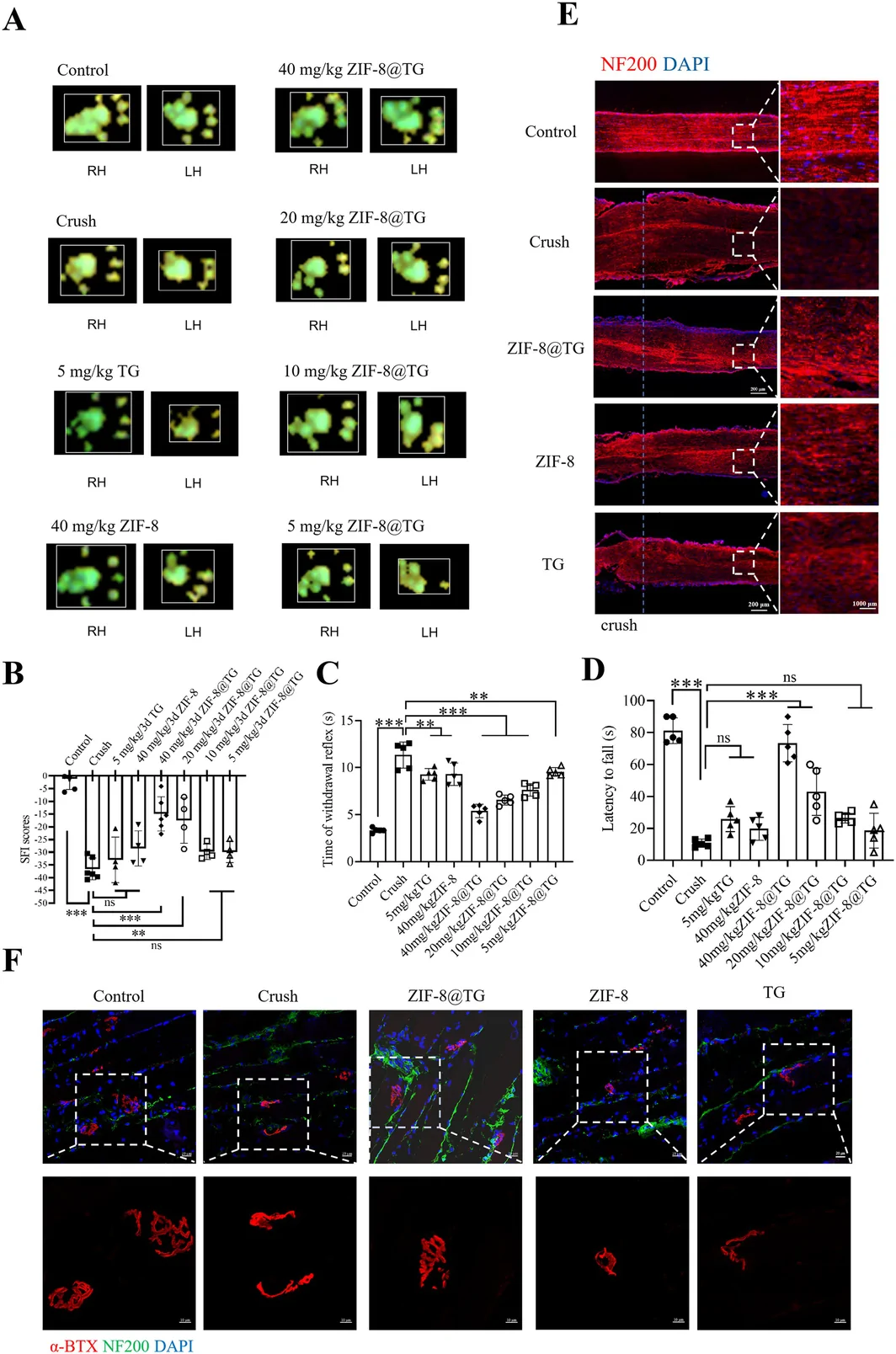
Effects of ZIF‐8@TG on motor function and motor nerves in mice with sciatic nerve crush. (A) Representative footprint images of the injured left hindpaw (LH) and healthy right hindpaw (RH). (B) Quantification of the sciatic functional index (SFI). (C) Results of the thermal sensitivity test (*n* = 5). (D) Results of the inverted grid test (*n* = 5). (E) Representative immunofluorescence staining for neurofilament 200 (NF‐200) in longitudinal sections of the sciatic nerve. *n* = 3, scale bars: 1000 and 200 μm. (F) Representative staining of motor endplates (α‐BTX) in the TA muscle. *n* = 3, scale bars: 20 and 10 μm. Data are presented as mean ± SD; ns = not significant, *p < 0.05, **p < 0.01, ***p < 0.001; *n* ≥ 3.

Following nerve injury, although damaged axons possess a capacity for regeneration, the slow rate of this process is often insufficient to counterbalance the rapid progression of skeletal muscle atrophy [6, 18]. Furthermore, the NMJs play critical roles in motor function, with their structural integrity and distribution being closely correlated with muscular contractility and functional recovery [19]. Based on the established optimal dosage of 40 mg/kg ZIF‐8@TG from our preliminary functional analyses, we further investigated its potential effects on injury‐associated neural structural alterations following injury. Immunofluorescence staining for neurofilament 200 (NF‐200) revealed that the crush group exhibited impaired axonal regeneration. In contrast, the ZIF‐8@TG treatment group showed a significant increase in regenerated axon length, outperforming both the ZIF‐8 and TG alone groups (Figure 3E), suggesting that ZIF‐8@TG is associated with improved axonal structural recovery after sciatic nerve crush injury. NMJ morphology analysis revealed that the number and area of NMJs in the crush group were significantly smaller than those in control group, suggesting NMJ degeneration following injury. After treatment, both parameters were improved, with the most significant recovery observed in the ZIF‐8@TG‐treated group (Figure 3F). These findings indicate that ZIF‐8@TG nanoparticles are associated with preservation of NMJ morphology following injury. Collectively, these results suggest that ZIF‐8@TG may contribute to structural improvements of axons and NMJs following injury.

### 3.4 ZIF‐8@TG Alleviates Skeletal Muscle Atrophy Caused by Sciatic Nerve Crush

Based on the dose‐dependent improvement in motor function observed with ZIF‐8@TG treatment, particularly at 40 mg/kg, we next sought to validate its efficacy in alleviating nerve crush–induced skeletal muscle atrophy. The analysis showed that sciatic nerve crush injury led to a significant reduction in TA muscle weight. Although all treatment groups exhibited some improvement in muscle mass loss, the ZIF‐8@TG administered group demonstrated a significantly higher TA muscle mass than ZIF‐8 or TG treatment group (Figure 4A). Subsequently, laminin staining further revealed that compared to the control group, the crush group exhibited a significant reduction in the mean CSA of myofibers along with a marked leftward shift in the myofiber size distribution (Figure 4B–D), indicating successful induction of TA muscle atrophy by sciatic nerve crush. In contrast, the ZIF‐8@TG treatment group demonstrated a significantly larger mean myofiber CSA than that in crush group as well as ZIF‐8 or TG treatment group. Moreover, the leftward shift in myofiber distribution was substantially suppressed following ZIF‐8@TG administration (Figure 4B–D). These results collectively indicate that ZIF‐8@TG can effectively alleviate sciatic nerve crush–induced skeletal muscle atrophy.

**FIGURE 4:**
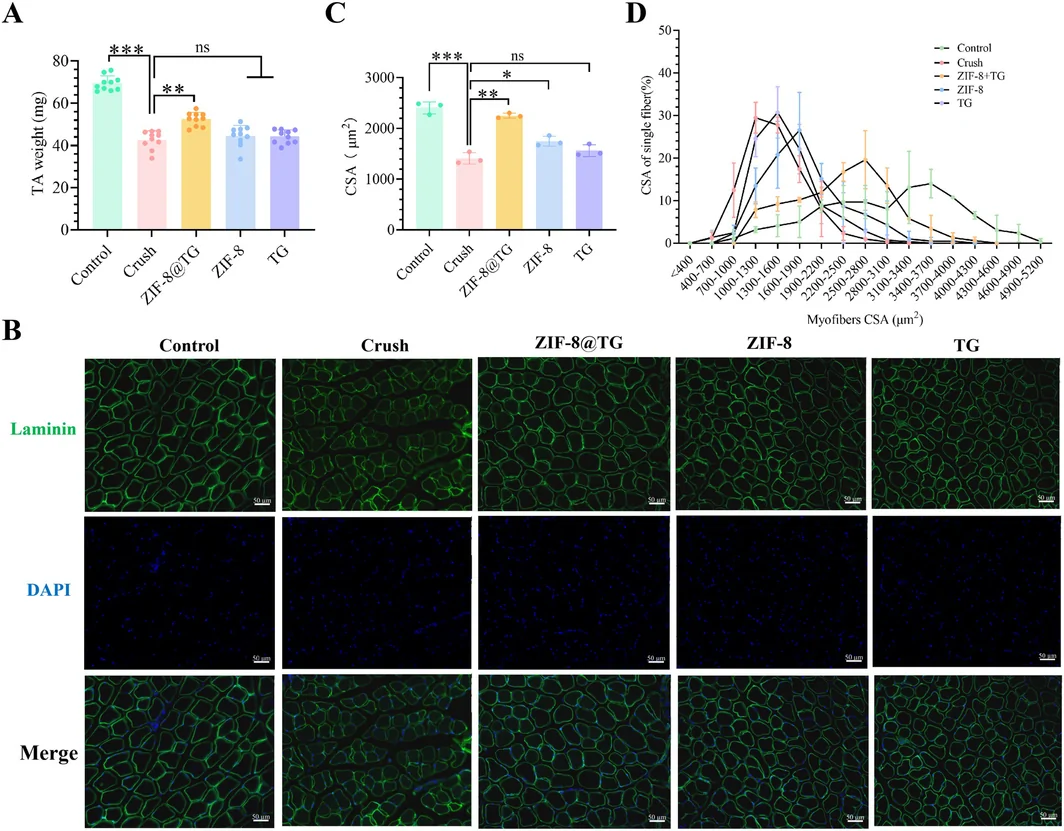
ZIF‐8@TG alleviates sciatic nerve crush‐induced atrophy in the mouse TA muscle. (A) Statistical analysis of TA wet weight (*n* = 10). (B) Representative immunofluorescence staining images of laminin showing the cross‐sectional area (CSA) of myofibers in the TA muscle across groups. Scale bar: 50 μm. (C) Quantitative analysis of the average myofiber CSA (*n* = 3). (D) Frequency distribution of myofiber CSAs. Data are presented as mean ± SD (*n* = 3); ns = not significant, **p < 0.01, ***p < 0.001.

### 3.5 ZIF‐8@TG Is Associated With Reduced Overactivation of the Ubiquitin‐Proteasome System and Modulates Autophagy‐Associated Alterations

During muscle atrophy following nerve injury, the homeostasis of the intramuscular environment is disrupted, leading to increased degradation of muscle proteins and suppressed protein synthesis [4]. Western blot showed a significant downregulation of MHC in crush group, indicating the TA muscle protein homeostasis is disrupted by sciatic nerve crush. Consistent with the in vitro findings (Figure 2), treatment with ZIF‐8@TG nanoparticles significantly attenuated the injury‐induced decrease in MHC protein levels, outperforming both the ZIF‐8 and TG treatment groups (Figure 5A,B). The UPS and ALP represent two major proteolytic systems involved in skeletal muscle atrophy. FoxO3a is a key transcription factor regulating both pathways. Western blot demonstrated that their levels were significantly upregulated in the crush group, whereas ZIF‐8@TG treatment markedly suppressed the increase in MuRF1, MAFbx and FoxO3a (Figure 5A,B). Collectively, these results suggest that ZIF‐8@TG nanoparticles are associated with reduced excessive muscle protein degradation, potentially via attenuation of the UPS system.

**FIGURE 5:**
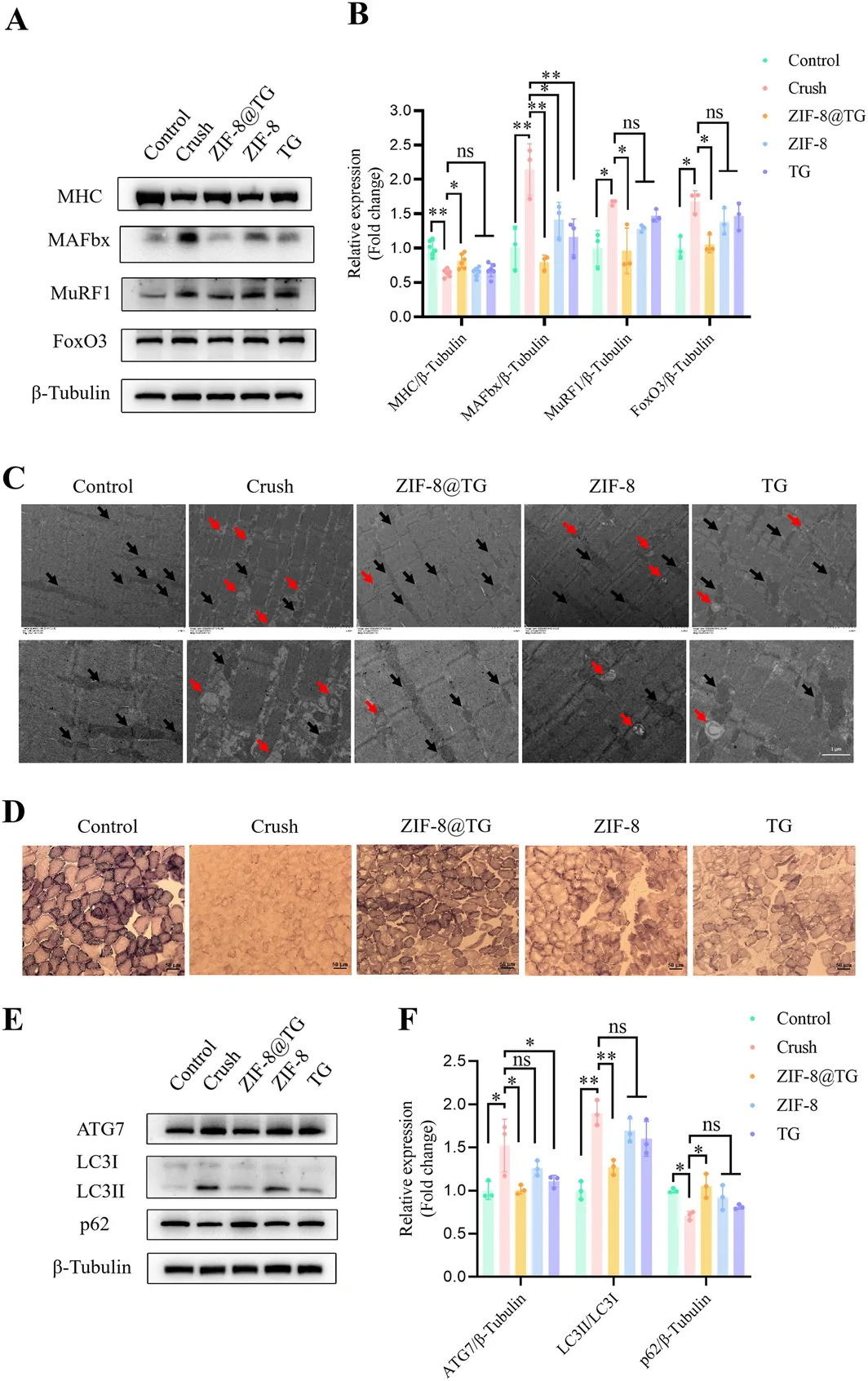
ZIF‐8@TG is associated with reduced overactivation of the ubiquitin‐proteasome system and autophagic lysosomal system during sciatic nerve crush‐induced muscle atrophy. (A) Western blot analysis of MHC, the E3 ubiquitin ligases MAFbx and MuRF1 and FoxO3a protein levels in the TA muscle. (B) The quantitative analysis of MHC (*n* = 6), MAFbx (*n* = 3), MuRF1 (*n* = 3) and FoxO3a (*n* = 3). (C) Representative TEM images of TA muscle tissue (magnified views below). Black arrows indicate normal mitochondria; red arrows indicate mitochondrial vacuoles and autophagosomes. *n* = 3, scale bar: 2 and 1 μm. (D) Representative images of succinate dehydrogenase (SDH) staining in TA muscle sections. *n* = 3, scale bar: 50 μm. (E) Western blot analysis of ATG7, LC3‐II and p62 protein levels in the TA muscle. (F) The quantitative analysis of ATG7 (*n* = 3), LC3‐II (*n* = 3) and p62 (*n* = 3) protein levels. Data are presented as mean ± SD; ns = not significant, *p < 0.05, **p < 0.01, ***p < 0.001.

Autophagy exerts a dual regulatory effect on skeletal muscle homeostasis. On one hand, basal autophagy contributes to cellular quality control by clearing damaged organelles and maintaining muscle homeostasis [6]; on the other hand, excessive ALP activation exacerbates atrophy [20, 21]. The TEM imaging revealed that the sciatic nerve crush group exhibited significant pathological changes, characterized by the presence of abundant mitochondrial vacuoles and autophagosomes, suggesting mitochondrial damage and altered autophagy‐associated activity. These pathological features were substantially alleviated after ZIF‐8@TG intervention (Figure 5C). The SDH staining showed that SDH activity was significantly reduced in the crush group, indicating severe impairment of mitochondrial function. In contrast, treatment with ZIF‐8@TG resulted in the most substantial recovery of SDH activity among all therapeutic groups, outperforming both ZIF‐8 and TG alone (Figure 5D), suggesting effective preservation of mitochondrial function during nerve injury–induced skeletal muscle atrophy. Western blot analysis further demonstrated that the crush group exhibited a marked downregulation of p62, a selective autophagy substrate receptor protein, along with significantly elevated levels of the key autophagy‐related proteins ATG7 and LC3 II, suggesting that substantial alterations in autophagy‐associated protein expression. The observed increase may reflect increased autophagic flux. Notably, treatment with ZIF‐8@TG significantly reversed these alterations, outperforming the effects observed in the ZIF‐8 or TG alone groups (Figure 5E,F). These findings suggest that ZIF‐8@TG is associated with autophagy‐associated changes during nerve injury–induced muscle atrophy.

### 3.6 ZIF‐8@TG Is Associated With Activation of the Protein Synthesis–Related Akt/mTOR Signalling Pathway in Skeletal Muscle

In addition to suppressing proteolytic pathways, restoring protein synthesis capacity represents a complementary therapeutic strategy against muscle atrophy [22, 23]. To investigate whether ZIF‐8@TG influences anabolic signalling related to protein synthesis, we examined the phosphorylation levels of key proteins in the Akt/mTOR pathway by western blot. The results showed that the levels of p‐Akt and p‐mTOR exhibited a marked downregulation in the crush group, suggesting suppression of Akt/mTOR signalling during nerve injury–induced skeletal muscle atrophy. ZIF‐8@TG treatment significantly elevated the levels of p‐Akt and p‐mTOR (Figure 6A–C), suggesting that ZIF‐8@TG is associated with activation of the protein synthesis–related Akt/mTOR signalling pathway during nerve injury–induced skeletal muscle atrophy.

**FIGURE 6:**
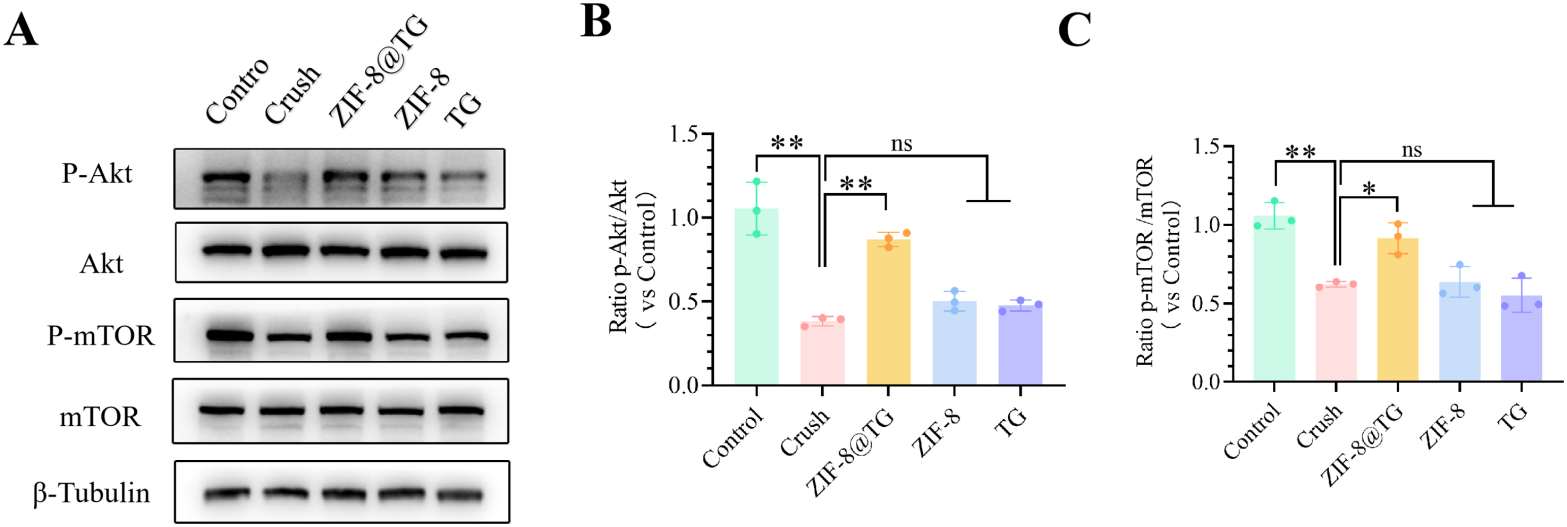
ZIF‐8@TG is associated with activation of protein synthesis during sciatic nerve crush‐induced muscle atrophy. (A) Western blot analysis of phosphorylated Akt (p‐Akt), Akt, phosphorylated mTOR (p‐mTOR) and mTOR protein levels in the TA muscle. (B) Quantitative analysis of the p‐Akt/Akt ratio (*n* = 3). (C) Quantitative analysis of the p‐mTOR/mTOR ratio (*n* = 3). Data are presented as mean ± SD; ns = not significant, *p < 0.05, **p < 0.01, ***p < 0.001.

### 3.7 ZIF‐8@TG Alleviates Oxidative Stress in Sciatic Nerve Crush–Induced Skeletal Muscle Atrophy

Oxidative stress, as an early upstream driver of protein homeostasis imbalance, plays a critical role in the initiation and progression of skeletal muscle atrophy [6]. Furthermore, our previous results demonstrated severe impairment of mitochondrial structure and function (Figure 5C), which may lead to excessive release of reactive oxygen species (ROS) and exacerbate mitochondrial damage. We therefore evaluated the effects of ZIF‐8@TG on counteracting oxidative stress during nerve crush‐induced muscle atrophy. ROS levels showed a significant increase by DHE probe in the TA muscle of the crush, whereas ZIF‐8@TG treatment markedly suppressed ROS accumulation, outperforming other treatment groups (Figure 7A,B). Furthermore, ZIF‐8@TG significantly suppressed the upregulation of pro‐oxidant enzymes NOX2 and NOX4 while restoring the expression of antioxidant enzymes SOD1 and GPX1 and the transcription factor Nrf2 (Figure 7C,D). These findings collectively show that ZIF‐8@TG treatment is associated with suppressed oxidative stress during nerve injury–induced skeletal muscle atrophy, with superior efficacy compared to ZIF‐8 or TG treatment.

**FIGURE 7:**
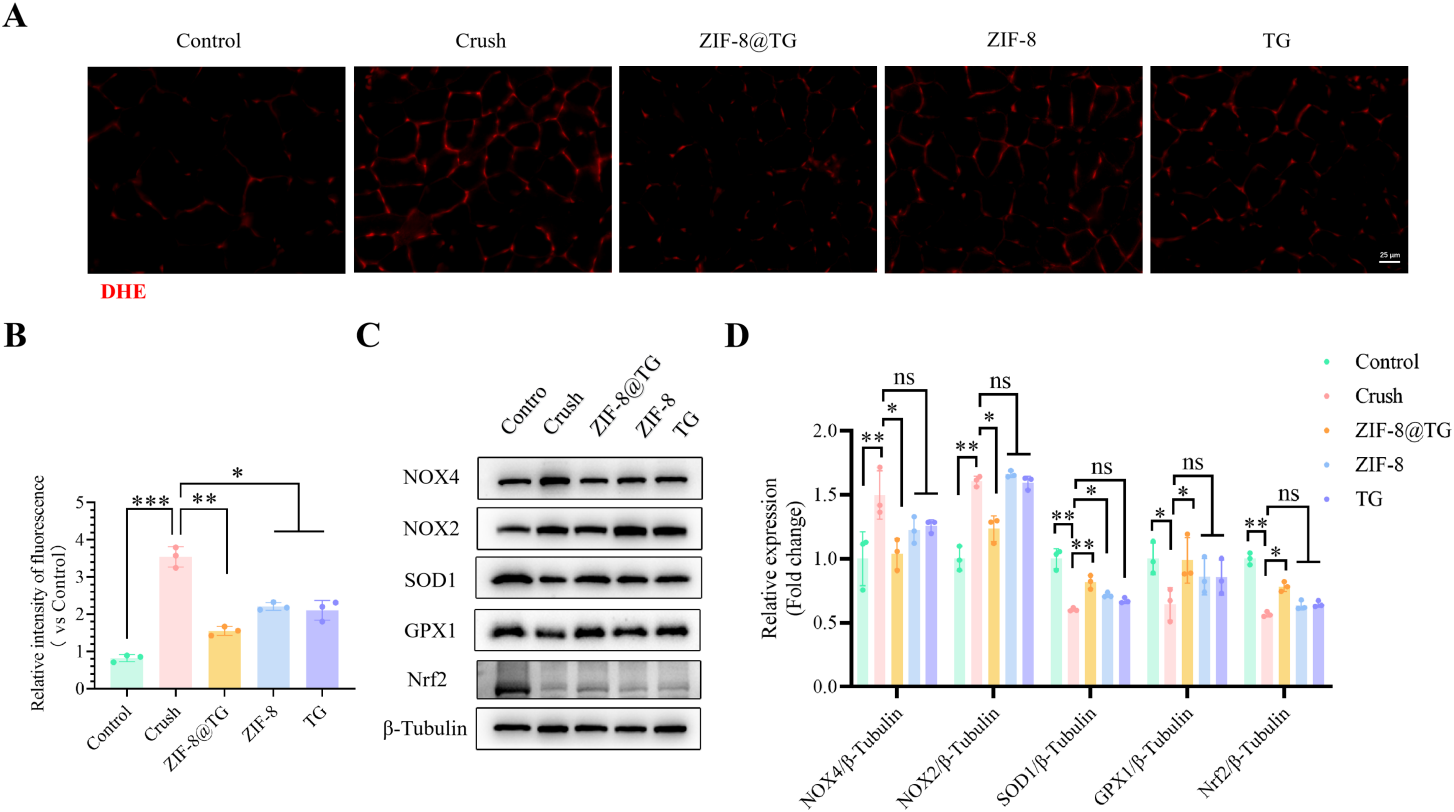
ZIF‐8@TG alleviates oxidative stress during skeletal muscle atrophy. (A) Representative images of DHE fluorescence staining in TA muscle sections. Scale bar: 25 μm. (B) Quantitative analysis of relative DHE fluorescence intensity (*n* = 3). (C) Western blot analysis of NOX4, NOX2, SOD1, GPX1 and Nrf2 protein levels in the TA muscle. (D) Quantification analysis of NOX4 (*n* = 3), NOX2 (*n* = 3), SOD1 (*n* = 3), GPX1 (*n* = 3) and Nrf2 (*n* = 3) protein levels. Data are presented as mean ± SD; ns = not significant, *p < 0.05, **p < 0.01, ***p < 0.001.

### 3.8 ZIF‐8@TG Alleviates Inflammation in Sciatic Nerve Crush–Induced Skeletal Muscle Atrophy

Inflammation and oxidative stress form a vicious cycle that exacerbates muscle atrophy [4]. H&E staining revealed marked inflammatory cell infiltration in the TA muscle after nerve crush, which was substantially reduced by ZIF‐8@TG treatment (Figure 8A). Immunofluorescence staining and western blot analysis of CD68 further revealed a significant increase in CD68 expression in the crush group, whereas ZIF‐8@TG treatment effectively suppressed this increase (Figure 8B–E), indicating that ZIF‐8@TG inhibits the infiltration of inflammatory cells, particularly macrophages. Western blot results demonstrated that the crush group exhibited significantly elevated levels of pro‐inflammatory cytokines, including IL‐1β, IL‐6 and TNF‐α. In contrast, ZIF‐8@TG treatment significantly suppressed the upregulation of these cytokines (Figure 8D,E). The nuclear factor kappa B (NF‐κB) is recognized as a central mediator of inflammation‐driven muscle atrophy [4]. In addition, IL‐6 activates the JAK/STAT3 pathway, which directly promotes the expression of *MAFbx* and *MuRF1* [24]. Western blot results demonstrated that the phosphorylation levels of NF‐κB and STAT3 in the crush group were significantly upregulated, suggesting that the NF‐κB and STAT3 pathways were activated. ZIF‐8@TG treatment markedly inhibited their activation, outperforming the ZIF‐8 or TG alone groups (Figure 8D,E). Taken together, these results suggest that suppression of inflammatory responses may contribute to the alleviation of skeletal muscle atrophy following nerve injury by ZIF‐8@TG, which is consistent with its multi‐target therapeutic potential.

**FIGURE 8:**
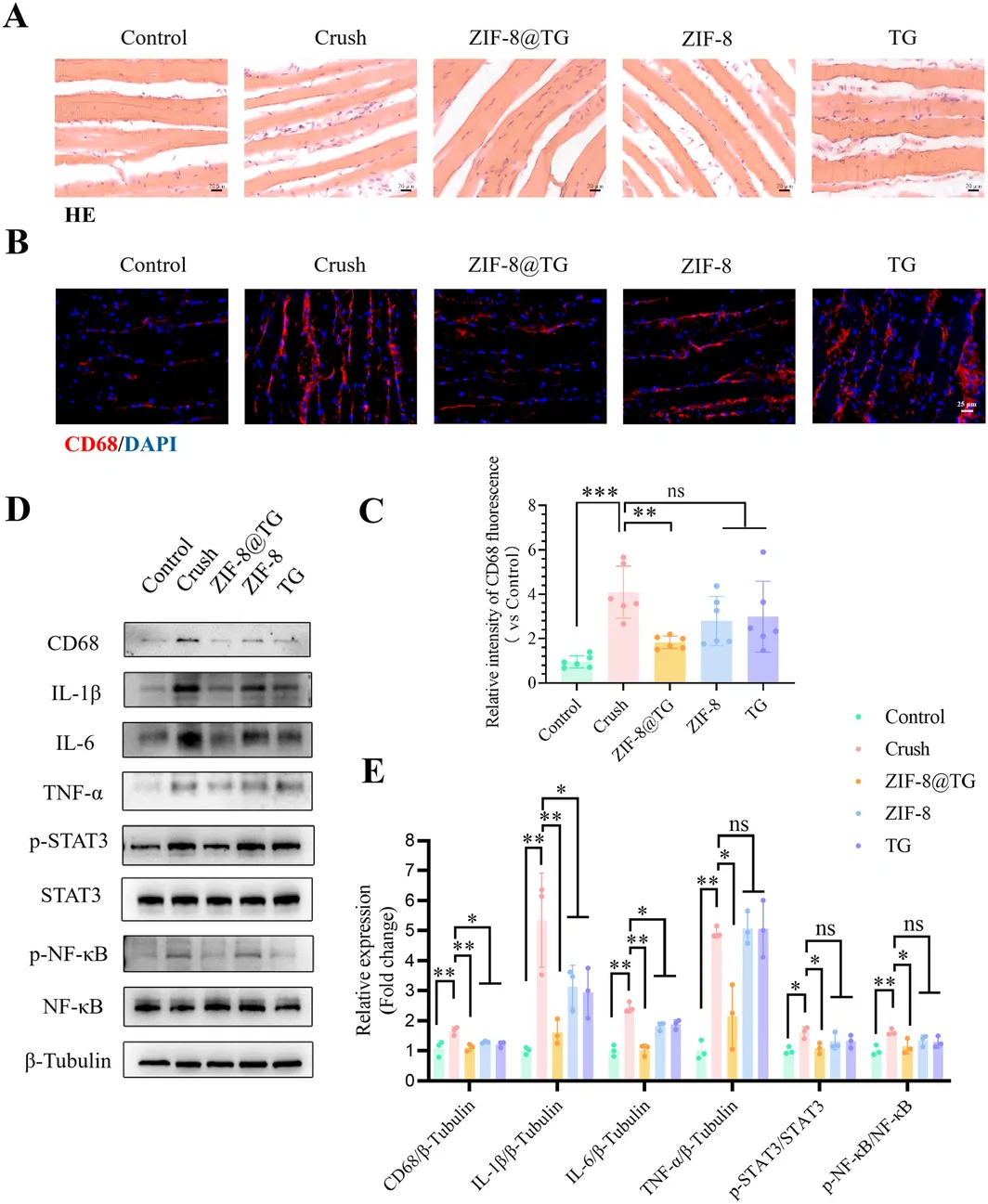
ZIF‐8@TG alleviates inflammation during skeletal muscle atrophy. (A) Representative H&E staining images of TA muscle sections. *n* = 3, scale bar: 20 μm. (B) Representative immunofluorescence images of CD68 in TA muscle sections. Scale bar: 25 μm. (C) Quantification of CD68 immunofluorescence intensity (*n* = 6). (D) Western blot analysis of CD68, IL‐1β, IL‐6, TNF‐α, phosphorylated STAT3 (p‐STAT3), STAT3, phosphorylated NF‐κB (p‐NF‐κB) and NF‐κB protein levels in the TA muscle. (E) Quantification analysis of CD68 (*n* = 3), IL‐1β (*n* = 3), IL‐6 (*n* = 3), TNF‐α (*n* = 3), p‐STAT3/STAT3 ratio (*n* = 3) and p‐NF‐κB/NF‐κB ratio (*n* = 3). Data are presented as mean ± SD; ns = not significant, *p < 0.05, **p < 0.01, ***p < 0.001.

## 4 Discussion

Neurogenic muscle atrophy results in functional impairment, yet effective interventions to halt its progression remain lacking [3, 25]. Compared with conventional pharmacological interventions, nanotherapeutic systems provide unique advantages, including improved drug stability, enhanced bioavailability, controlled release behaviour and targeted delivery to injured tissues. The metal–organic frameworks, particularly ZIF‐8‐based nanodelivery systems, offer a promising platform for developing novel therapeutic approaches [14]. This study demonstrated that ZIF‐8@TG effectively improved muscle function and mitigated the decline of muscle mass in mice of sciatic nerve crush–induced muscle atrophy, effects that may be related to restoration of proteostasis, potentially involving its antioxidant and anti‐inflammatory properties. The ZIF‐8@TG is associated with direct protection of muscle as well as structural improvements of axons and NMJs following injury. Our findings support that nanotechnology‐based therapeutic strategies may represent a promising direction for skeletal muscle atrophy treatment.

Recent advances in nanomedicine for skeletal muscle disorders have demonstrated the potential of various nanoplatforms, including metal–organic frameworks, liposomes, polymeric nanoparticles and two‐dimensional nanomaterials, to address the complex pathophysiology of muscle atrophy [26]. Liposomal systems enable co‐delivery of bioactive agents to mitigate oxidative stress and preserve mitochondrial function in in vitro model of sarcopenia [27]. The self‐powered nanofibre membranes loaded with anti‐inflammatory agents [28] and extracellular vesicle–loaded hydrogels [29] have demonstrated efficacy in denervation‐induced muscle atrophy. ZIF‐8 serves as a versatile nanoplatform that demonstrates remarkable enhanced therapeutic effects in the treatment of various diseases, attributable to its structural characteristics and functional design [15]. For instance, ZIF‐8‐based SOD&Fe_3_O_4_@ZIF‐8 nanozymes achieve ROS scavenging and anti‐inflammatory effects [30]. In cancer, the encapsulation of mitoxantrone within ZIF‐8 improves tumour targeting and immunogenic cell death [31]. By integrating its carrier functionality, protection of active ingredients, capacity for responsive release and intrinsic bioactivity, ZIF‐8 significantly enhances therapeutic outcomes across multiple disease models. Importantly, in our study, ZIF‐8@TG demonstrated substantially enhanced efficacy in alleviating sciatic nerve crush–induced skeletal muscle atrophy compared to either TG or ZIF‐8 alone. The improved efficacy of ZIF‐8@TG may be attributed to multiple factors. First, encapsulation within ZIF‐8 likely enhances the stability and bioavailability of TG. Second, the ZIF‐8 carrier itself may contribute through its intrinsic bioactivity, such as ROS‐scavenging capacity, or by improving tissue retention. Third, a combination of enhanced delivery and carrier effects may act together. Future studies will be necessary to dissect these contributions. Moreover, the tissue‐targeting specificity of ZIF‐8@TG towards injured skeletal muscle remains relatively limited. The precise in vivo release kinetics and biodistribution profile of TG after encapsulation within ZIF‐8 have not yet been fully characterized. These issues may affect therapeutic efficiency and translational applicability and therefore warrant further investigation.

Skeletal muscle is the largest organ in the human body [32, 33]. The molecular mechanisms underlying skeletal muscle atrophy are highly complex, with the core pathology being a disruption in proteostasis. On one hand, protein synthesis signalling pathways such as Akt/mTOR are suppressed; on the other hand, proteolytic systems are markedly activated [4]. Our results show that ZIF‐8@TG treatment is associated with suppressed upregulation of FoxO3a and key proteins in the UPS and ALP during sciatic nerve crush‐induced muscle atrophy. Concurrently, ZIF‐8@TG was associated with partially counteraction of the inhibition of the Akt/mTOR signalling pathway. Together, these effects attenuate the decrease in MHC levels, help preserve muscle proteostasis and delay the progression of skeletal muscle atrophy. These findings are consistent with previous studies suggesting that restoration of proteostasis is a critical therapeutic strategy in neurogenic muscle atrophy and further indicate that ZIF‐8 nanocarrier‐mediated TG delivery may enhance the efficacy of bioactive compounds in regulating catabolic signalling pathways.

Oxidative stress and inflammation serve as critical upstream drivers of the UPS and ALP, promoting proteolysis and suppressing protein synthesis, thereby playing a central role in the initiation and progression of muscle atrophy [6, 34]. Oxidative stress is widely recognized as an early key event triggering skeletal muscle atrophy [6, 35]. When the production of free radicals exceeds the body's antioxidant defence capacity, excess ROS can directly attack myofibrillar proteins, such as MHC, leading to structural alterations and functional impairment. As an antioxidant, TG can upregulate the levels of antioxidant enzymes to scavenge ROS [9]. In the present study, we found that ZIF‐8@TG loaded with TG was associated with the reduction of ROS during sciatic nerve crush–induced muscle atrophy. Inflammation also plays an equally critical role in the initiation and maintenance of skeletal muscle atrophy [4]. Under various pathological conditions such as nerve injury, unloading and chronic diseases, local or systemic inflammatory responses become activated. Moreover, a vicious cycle often forms between inflammation and oxidative stress, thereby mutually exacerbating skeletal muscle atrophy. Immune cells infiltrate muscle tissue and release pro‐inflammatory cytokines [4, 36]. These cytokines bind to receptors, activating key downstream signalling pathways such as STAT3 and NF‐κB, which in turn amplify the production of inflammatory mediators and perpetuate the inflammation [4]. Our study shows that ZIF‐8@TG treatment is associated with suppressed inflammatory cell infiltration, downregulated levels of TNF‐α, IL‐1β and IL‐6 during sciatic nerve crush–induced muscle atrophy. These findings collectively suggest that ZIF‐8@TG treatment is associated with alleviation of inflammation and oxidative stress during sciatic nerve crush–induced skeletal muscle atrophy, which may help restore proteostasis in skeletal muscle.

Despite the supportive evidence described above, it is important to acknowledge that most of our mechanistic conclusions are based on marker‐level associations. A key mechanistic question that remains unresolved concerns the downstream signalling pathways through which ZIF‐8@TG exerts its anti‐atrophy effects. Previous work has identified PKACα as a direct molecular target of TG in skeletal muscle, where TG promotes GLUT4 expression and improves insulin resistance through activation of the PKA/AMPK signalling axis [10]. Given that PKA can function upstream of AMPK and that AMPK is a well‐established regulator of muscle protein metabolism and proteostasis [37], it is plausible that the PKA/AMPK pathway may also contribute to the anti‐atrophy effects of ZIF‐8@TG observed in the present study. In future studies, genetic methods such as knockout/knock‐in of AMPKα or PKA, pharmacological inhibition and rescue experiments can be employed to conduct the final causal verification in order to explore the roles of pathways like PKA/AMPK in the anti‐atrophy effect of ZIF‐8@TG. Furthermore, it is important to note that the sciatic nerve crush model primarily represents neurogenic muscle atrophy caused by denervation. Although this model shares several core pathophysiological features with other forms of muscle wasting, including oxidative stress, inflammation, and proteolytic activation, the underlying aetiology and dominant mechanisms may differ. Therefore, the protective effects of ZIF‐8@TG in other types of muscle wasting remain unknown and require further validation in clinically relevant models such as aged mice, cancer cachexia or disuse atrophy.

In summary, ZIF‐8@TG effectively alleviates sciatic nerve crush–induced muscle atrophy and offers a promising nanotherapeutic strategy. However, the current biosafety evaluation was limited to short‐term observation; comprehensive assessment of long‐term toxicity, pharmacokinetics and chronic administration safety is required before clinical translation can be considered. Furthermore, optimizing particle size, targeting efficiency and drug‐loading stability may improve the therapeutic performance of ZIF‐8@TG. Comparative studies with other nanoplatforms, validation in additional atrophy models and comprehensive long‐term safety assessment will be valuable for supporting its clinical translation.

## Funding

This work was funded by the National Natural Science Foundation of China (Nos. 82401633, 32130060, 82072160 and 82673254), the Basic Research Program of Jiangsu (No. BK20232023), the Natural Science Research Projects in Universities of Jiangsu Province (Nos. 24KJA310007 and 24KJB310013), Suzhou Science and Technology Development Program (No. SYW2024048) and Nantong Science and Technology Program (No. 20241118084).

## Conflicts of Interest

The authors declare no conflicts of interest.

## Supporting information


**Data S1:** Supporting Information.

**Figure S1:** (A) X‐ray diffraction (XRD) patterns of ZIF‐8 and ZIF‐8@TG nanoparticles. (B) Zeta potential of ZIF‐8 and ZIF‐8@TG nanoparticles. (C) Dynamic light scattering (DLS) of ZIF‐8 and ZIF‐8@TG nanoparticles. (D) Polydispersity index (PI) of ZIF‐8 and ZIF‐8@TG nanoparticles. (E) Stability of ZIF‐8@TG nanoparticles in normal saline.

**Figure S2:** (A) In vivo imaging of mice at 1 and 24 h after injection of Cy5‐TG and Cy5‐ZIF‐8@TG. The injured muscles were marked by white circles. (B) Ex vivo images of injured muscles at 24 h after injection.

**Figure S3:** In vivo safety evaluation of ZIF‐8@TG. Representative haematoxylin and eosin (H&E) stained sections of the heart, liver, spleen, lung and kidney from mice following treatment with ZIF‐8@TG, ZIF‐8 or TG. Scale bar: 100 μm.

## Data Availability

Data will be made available on request.
