# Supplementary material for: ZIF‐8‐Based Nanodelivery of Tectorigenin for Alleviating Skeletal Muscle Atrophy via Inhibition of Oxidative Stress and Inflammation
Source: J Cachexia Sarcopenia Muscle. 2026 Nov 10;17(6):e70418. doi: 10.1002/jcsm.70418 (PMC13749619; doi:10.1002/jcsm.70418)
Supplement: Supplementary file 1 [file JCSM-17-e70418-s003.docx]

**ZIF-8-based nanodelivery of tectorigenin for alleviating skeletal muscle atrophy via inhibition of oxidative stress and inflammation**

**Authors:** Xia Li^1,2#^, Yutong Wei^1#^, Lei Qi^1,2#^, Xiu Dai^1#^, Zhanzhan Li^1^, Chuli Zhu^1^, Xucheng Zhu^1^, Yuntian Shen^1^, Bingqian Chen^3^, Xiaosong Gu, Hualin Sun^1*^, Xinlei Yao^1*^

1. Jiangsu Key Laboratory of Tissue Engineering and Neuroregeneration, Key Laboratory of Neuroregeneration of Ministry of Education, Co-innovation Center of Neuroregeneration, Medical School of Nantong University, Nantong University, Nantong, Jiangsu Province 226001, People's Republic of China
2. Department of Emergency Medicine, Affiliated Hospital of Nantong University, Nantong, Jiangsu Province 226001, People's Republic of China.
3. Department of Orthopedics, Changshu Hospital Affiliated to Soochow University, First People's Hospital of Changshu City, Changshu, Jiangsu Province 215500, People's Republic of China

\# These authors contributed equally to this work

**Corresponding author**:

[**sunhl@ntu.edu.cn**](mailto:sunhl@ntu.edu.cn) (Hualin Sun) and [**Xinlei_yao@ntu.edu.cn**](mailto:Xinlei_yao@ntu.edu.cn) (Xinlei Yao).

Jiangsu Key Laboratory of Tissue Engineering and Neuroregeneration, Nantong University. Jiangsu Province 226001, People's Republic of China

**Material and Methods**

**Reagents**: Fetal Bovine Serum was purchased from Sigma (Missouri, USA). Horse Serum and Trypsin were purchased from Gibco (Grand Island, USA). Dulbecco's Modified Eagle Medium (DMEM) and PBS buffer were purchased from Bio-channel Biotechnology (Nanjing, China). Penicillin-Streptomycin Solution was purchased from Beyotime Biotechnology (Shanghai, China). Hank's Balanced Salt Solution HBSS (B410KJ) was purchased from Basal Media (Shanghai, China). Primary antibodies against NF-κB (ab16502), phospho-NF-κB (ab86299), CD68 (ab125212), GPX1 (ab22604), Fbx32 (ab168372), and β-Tubulin (ab6406) were purchased from Abcam (Cambridge, UK). STAT3 (30835S), p-STAT3 (9145S), TNF-α (11948S), IL-1β (12426S), Akt (9272S), phospho-Akt (s473) (9271S), and FoxO3a (12829S) were purchased from Cell Signaling Technology (Massachusetts, USA). SOD1 (F1214), mTOR (F0169), phospho-mTOR (P18P17), ATG7 (F0320), and P62 (F0106) were purchased from Sellect (Texas, USA). IL-6 (26404-1-AP) and NOX2 (19013-1-AP) were purchased from Proteintech (Wuhan, China). CD68 (38005) and MuRF1 (38580) were purchased from Signalway Antibody (Maryland, USA). Nrf2 (YM8624) was purchased from Immunoway (Texas, USA). NOX4 (MA5-32090) was purchased from Invitrogen (USA). MHC (MAB4470) was purchased from R&D Systems (Minnesota, USA). LC3 (M186-3) was purchased from MBL (Tokyo, Japan). α-BTX (00007-100) was purchased from Biotium (California, USA). Donkey Anti-Rabbit IgG H&L Alexa Fluor® 488 (ab150073), Goat Anti-Mouse IgG H&L Alexa Fluor® 488 (ab150113), and Goat Anti-Mouse IgG H&L (Alexa Fluor® 594) (ab150116) were purchased from Abcam. DHE Probe (S0063), Hematoxylin-Eosin Staining Kit (C0105S), Skim Milk Powder (P0216-1500g), Immunostaining Blocking Solution (P0102), Anti-Fluorescence Quenching Mounting Medium (P0126-25), and BeyoBCA Protein Concentration Rapid Assay Kit (P0398M) were purchased from Beyotime Biotechnology. RIPA Lysis Buffer (PC101), Protease Inhibitor Cocktail (100×, without EDTA) (GRF101), and Phosphatase Inhibitor Cocktail (100×) (GRF102) were purchased from Epizyme Biomedical Technology (Shanghai, China). Pre-stained Protein Marker (26617) was purchased from Thermo Scientific (Massachusetts, USA). Succinate Dehydrogenase Staining Kit (G200) was purchased from Solarbio (Beijing, China). ECL Chemiluminescent Substrate (180-5001) was purchased from Tanon (Shanghai, China).

**Synthesis of ZIF-8 and ZIF-8@TG:** Briefly, a 5 mL methanolic solution of 0.5 M Zn(NO₃)₂·6H₂O (solution A) and a 5 mL methanolic solution of 5.55 M 2-methylimidazole (solution B) were prepared. The two solutions were then rapidly mixed and incubated in a shaker incubator at 100 rpm for 24 hours at room temperature. The resulting white precipitate was collected by centrifugation, washed, and subsequently dried in an oven at 60 °C. For the preparation of TG-loaded ZIF-8 (ZIF-8@TG), solutions A and B were first mixed and stirred magnetically for 8 minutes to allow pre-nucleation. Subsequently, TG powder was added to the mixture to achieve a final concentration of 10 mg/mL. After thorough mixing, the reaction proceeded under continuous shaking at 100 rpm for 24 hours. The resulting product was collected by centrifugation, washed thoroughly with deionized water twice to remove any unreacted precursors or free TG, and finally obtained as a light-yellow powder after freeze-drying. To evaluate the stability in normal saline, 0.2 mg of ZIF-8@TG nanoparticles were dispersed in normal saline and incubated at 37 °C with shaking. At 1 h and 24 h, aliquots were collected, ultrasonically dispersed, centrifuged, washed once with deionized water, and re‑dispersed in ethanol for SEM observation.

**Encapsulation efficiency**: The encapsulation efficiency (EE%) of TG in ZIF-8 nanoparticles was determined by ultraviolet-visible spectrophotometry. The supernatant obtained after centrifugation was analyzed using the preset standard calibration curve to quantitatively determine the concentration of unencapsulated TG. The encapsulation efficiency was calculated according to the following formula:

EE%=W_TG_/W_TG0_×100%

Drug loading capacity: The drug loading capacity (LC%) is calculated as the percentage of the encapsulated TG mass to the total mass of the freeze-dried nanoparticles. The content of encapsulated TG is determined by the mass balance method of the supernatant, while the total mass of the nanoparticles is the constant weight of the freeze-dried particles. The LC% is calculated using the following formula:

LC%= W_TG_/W_ZIF-8@TG_×100%

**Synthesis of Cy5-labeled TG (Cy5-TG) and Cy5-labeled ZIF-8@TG (Cy5-ZIF-8@TG)**: Briefly, Cy5 solution (400 μL, 10 mg/mL) was activated with EDC·HCl and NHS (58.8 μL each, 100 mM) at room temperature in the dark for 30 min. Then, 20 mg of TG or ZIF‑8@TG was added, ultrasonicated in an ice‑water bath for 15 min, and incubated at 150 rpm for 4 h at room temperature in the dark. For Cy5‑TG, the reaction was cooled at 4 °C, precipitated with 4 mL of ice‑water, centrifuged, washed twice with ice‑water, and freeze‑dried. For Cy5‑ZIF‑8@TG, the product was collected by ultrafiltration centrifugation (8000 rpm, 10 min), washed twice with anhydrous DMSO and twice with PBS (pH 7.4), freeze‑dried, and stored at −20 °C in the dark. All procedures were performed under light‑protected conditions to prevent Cy5 fluorescence quenching.

**Cy5-TG and Cy5-ZIF-8@TG in vivo biodistribution:** The in vivo biodistribution and injured muscle aggregation of Cy5-labelled free TG and Cy5-labelled ZIF-8@TG nanoparticles were investigated after intraperitoneal injection using an in vivo imaging system. After 18 days of the sciatic nerve crush surgery, the mice were randomly divided into two groups (n = 3) and injected with Cy5-TG and Cy5-ZIF-8@TG at a dose of 40 mg·kg⁻¹. Subsequently, the fluorescence signals were observed in an in vivo imaging system (IVIS, Caliper Life Sciences Inc., CA, USA) at 1h and 24h after injection, respectively. To further investigate the distribution of nanoparticles in muscles, mice were dissected at 24 h. The tibialis anterior muscle and gastrocnemius muscle were extracted to be photographed and imaged using an IVIS.

**Biosafety Assessment:** After 18 days of treatment with ZIF-8@TG, the mice were euthanized. Major organs, including the heart, liver, spleen, lungs, and kidneys, were collected and subjected to hematoxylin and eosin (H&E) staining for histological analysis. Briefly, following embedding in paraffin, tissue sections were cut at a thickness of 6 μm. The sections were then deparaffinized by immersion in xylene for 10 minutes. Subsequently, they were rehydrated through a graded ethanol series: sequential incubation in 100% ethanol, 95% ethanol, 85% ethanol, 70% ethanol, and pure water, for 5 minutes each. After rehydration, the sections were stained according to the manufacturer's instructions for the Hematoxylin and Eosin (H&E) Staining Kit.

**Preparation and Analysis of Release Samples**: A standard stock solution was prepared by dissolving 2 mg of the standard compound in PBS within a 20 mL volumetric flask to achieve a concentration of 100 µg/mL. This stock solution was then sequentially diluted with PBS to obtain a series of standard solutions with concentrations of 100, 50, 25, 12.5, 6.25, 3.125, and 0 µg/mL. The absorbance of these solutions was measured at a wavelength of 266 nm using a microplate reader (Bio-Tek, Eons, USA). A standard calibration curve was constructed by plotting the absorbance against the corresponding concentration. Dispersions of ZIF-8@TG (concentration: 5 mg/mL) were prepared in PBS at two different pH values (6.0 and 7.4). The dispersions were placed in a constant temperature shaker incubator maintained at 37 °C with continuous agitation. At predetermined time intervals (0.5, 1, 2, 3, 6, 12, 24, 48 and 72 hours), aliquots of the dispersions were centrifuged. Subsequently, 1 mL of the supernatant was carefully collected and replaced with an equal volume of fresh PBS to maintain a constant volume, after which the remaining solid was re-dispersed. The collected supernatant samples were stored at -20 °C until further analysis. After the completion of the release experiment, all frozen supernatant samples were thawed at 4 °C. The absorbance of each sample was then measured at 266 nm using the microplate reader. The cumulative drug release at each time point was calculated based on the previously established standard calibration curve.

**Cell culture:** C2C12 cells (within 15 passages, RRID: CVCL_0188) were cultured in complete medium containing DMEM and 10% FBS. When the C2C12 cell density reached 100%, the complete medium was abandoned, rinsed once with PBS buffer, and the differentiation medium containing DMEM and 2% horse serum was added. After changing the differentiation medium for 6 days, C2C12 cells differentiated into myotubes. Differentiated myotubes were incubated with HBSS containing either 20 μg/mL of ZIF-8@TG, ZIF-8, or TG, or with HBSS alone for 12 hours to assess their effects on nutrient deprivation-induced myotube atrophy. All of the cells were maintained under standard conditions (in a 37 ℃ incubator with 5% CO_2_) in the presence of 1% penicillin-streptomycin solution.

**Intracellular Uptake of ZIF8@TG:** C2C12 cells were seeded in small dishes, and after culture adherence, 20 μg/mL of ZIF-8@TG was added to culture for 6h. The culture medium was discarded, and the cells were washed 3 times with cold PBS to remove extracellular particles and digested with trypsin. The digestion was then terminated, and the cells were collected in a 15 ml centrifuge tube. After washing 3 times with PBS, 1 ml glutaraldehyde was added and fixed at 4°C for 2h. Subsequently, glutaraldehyde was discarded, 1% AGAR solution was added, and the clumps were removed and placed in glutaraldehyde when allowed to solidify. After sample preparation, the cell was observed by transmission electron microscopy.

**Animals and sciatic nerve crush models:** All experimental procedures strictly adhered to the animal care guidelines of Nantong University and were approved by the Jiangsu Provincial Experimental Animal Management Committee (approval No. S20240225-006). Adult male ICR mice **(**8-week, 30-32g, SPF) were provided by the Experimental Animal Center of Nantong University. Before the experiment, mice were acclimatized (60% humidity, 26°C, 12 h light-dark cycle) for 1 week. Mice were randomly assigned to each experimental group. After anesthesia with 1.25% avertin, a skin incision of about 2 cm in length was made along the femur of the mice, and the muscles were bluntly separated, and the sciatic nerve was exposed. Using hemostatic forceps with a 3-mm tip, the sciatic nerve was clamped for 15 seconds, and this procedure was repeated twice. After releasing the forceps, the clamped segment of the nerve became transparent. Post-surgery, the wound was routinely sutured, and the animals were maintained under standard housing conditions. The treatment groups were administered intraperitoneal injections every three days with the following: 5 mg·kg⁻¹ TG, 40 mg·kg⁻¹ ZIF-8, 5 mg·kg⁻¹ ZIF-8@TG, 10 mg·kg⁻¹ ZIF-8@TG, 20 mg·kg⁻¹ ZIF-8@TG, and 40 mg·kg⁻¹ ZIF-8@TG. Notably, as determined by our drug loading efficiency, the amount of TG loaded in the 40 mg·kg⁻¹ ZIF-8@TG dose is approximately equal to that in the 5 mg·kg⁻¹ free TG dose. The remaining groups were administered the vehicle solution of 10% DMSO in normal saline. The investigators performing behavioral tests and data analysis were blinded to the group allocation. Tissue samples were collected for analysis 18 days post-treatment.

**Western Blot:** Fresh tibialis anterior muscle tissues were homogenized in lysis buffer supplemented with phosphatase and protease inhibitors. The homogenates were lysed on ice for 30 min, followed by centrifugation at 11,000 rpm for 10 min at 4 °C. The supernatant was collected, and protein concentration was determined using a standard BCA assay. Protein samples were prepared to a final concentration of 4  μg/μL. Proteins were separated by SDS-PAGE and then transferred onto a PVDF membrane using a wet transfer system at 110 V for 60 min. After transfer, the membrane was blocked with blocking buffer at room temperature for 2 h with gentle shaking. The membrane was then washed three times with TBST and incubated with primary antibody at 4 °C overnight. Following primary antibody incubation, the membrane was washed three times with TBST (10  min per wash), incubated with HRP-conjugated secondary antibody at room temperature for 1 h with shaking, and washed again three times with TBST (10  min per wash). Protein bands were visualized using an enhanced chemiluminescence substrate by chemiluminescence imaging system, and the results were quantified using ImageJ software.

**Immunofluorescence Staining:**

For cells, after being washed three times with PBS, they were fixed with 4% paraformaldehyde for 20 min at room temperature. Following three PBS washes, the cells were permeabilized with 0.2% Triton X-100 for 10 min at room temperature and then blocked with 5% BSA for 30 min at room temperature. After the removal of BSA, the cells were incubated with the primary antibody at 4°C overnight. The next day, the primary antibody was discarded, and the cells were washed three times with PBS. Subsequently, they were incubated with a fluorochrome-conjugated secondary antibody in the dark for 1 h at room temperature. After removing the secondary antibody and performing three additional PBS washes, the cells were mounted with an antifade mounting medium containing DAPI. For muscle tissue, fresh tibialis anterior muscles were fixed in 4% paraformaldehyde for 24 h, followed by sequential dehydration in 10%, 20%, and 30% sucrose solutions. The tissues were then embedded in OCT compound and frozen at -20°C. Sections of 6 μm thickness were prepared by freezing microtome. The sections were washed three times with PBS (10 min per wash), blocked with immunofluorescence blocking buffer for 1 h at room temperature, and then incubated with the primary antibody at 4°C overnight. After washing three times with PBS (10 min per wash), the sections were incubated with a fluorochrome-conjugated secondary antibody in the dark for 2 h at room temperature. Finally, following three additional PBS washes (10 min per wash), the sections were mounted with an antifade mounting medium containing DAPI. Muscle sections were obtained from the mid‑belly of the tibialis anterior at comparable levels, two random fields per section from three animals per group were analyzed.

**Tests for thermal sensitivity:** Each mouse was individually placed on the surface and allowed to acclimate for at least 30 minutes. After the mouse became calm and stationary, the heat source generator was moved to the plantar center of hind paw. The heat source was then activated simultaneously with the start of the timer. The stimulus was immediately terminated upon a rapid withdrawal, licking, or shaking of hind paw, with the latency to this response recorded as the withdrawal reflex latency. If no response occurred within 30 seconds, the stimulus was automatically stopped and the latency was recorded as 30 seconds. Each hind paw was typically tested three times, with an interval of at least 15 minutes between consecutive measurements. The average of the measurements was calculated as the final value for each mouse.

**Inverted Grid Test:** The inverted grid consisted of a 10 × 14 cm metal wire grid with a wire diameter of 2 mm, positioned 40 cm above a protective surface. Each mouse was placed at the center of the grid, which was then smoothly inverted. The time until the mouse released its grip and fell was recorded, with a maximum time of 90 seconds. Each mouse underwent three trials with an interval of at least 10 minutes, and the best performance among the three trials was used as the final result for analysis.

**Supplementary Figures**

Supplementary Figure 1: (A) X-ray diffraction (XRD) patterns of ZIF-8 and ZIF-8@TG nanoparticles. (B) Zeta potential of ZIF-8 and ZIF-8@TG nanoparticles. (C) Dynamic Light Scattering (DLS) of ZIF-8 and ZIF-8@TG nanoparticles. (D) Polydispersity Index (PI) of ZIF-8 and ZIF-8@TG nanoparticles. (E) Stability of ZIF-8@TG nanoparticles in normal saline.

Supplementary Figure 2. (A) In vivo imaging of mice at 1h and 24h after injection of Cy5-TG and Cy5-ZIF-8@TG. The injured muscles were marked by white circles. (B) Ex vivo images of injured muscles at 24 h after injection.

Supplementary Figure 3: In vivo safety evaluation of ZIF-8@TG. Representative hematoxylin and eosin (H&E) stained sections of the heart, liver, spleen, lung, and kidney from mice following treatment with ZIF-8@TG, ZIF-8, or TG. Scale bar: 100 μm.
